# A SNP panel and online tool for checking genotype concordance through comparing QR codes

**DOI:** 10.1371/journal.pone.0182438

**Published:** 2017-09-19

**Authors:** Yonghong Du, Joshua S. Martin, John McGee, Yuchen Yang, Eric Yi Liu, Yingrui Sun, Matthias Geihs, Xuejun Kong, Eric Lingfeng Zhou, Yun Li, Jie Huang

**Affiliations:** 1 School of Statistics, Beijing Normal University, Beijing, China; 2 Department of Genetics, University of North Carolina Chapel Hill, Chapel Hill, North Carolina, United States of America; 3 NC Translational and Clinical Sciences Institute, University of North Carolina Chapel Hill, Chapel Hill, North Carolina, United States of America; 4 Department of Computer Science, University of North Carolina Chapel Hill, Chapel Hill, North Carolina, United States of America; 5 Department of Mathematics & Statistics, Boston University, Boston, Massachusetts, United States of America; 6 Department of Computer Science, Technische Universität Darmstadt, Darmstadt, Germany; 7 Beth Israel Deaconess Medical Center, Boston, Massachusetts, United States of America; 8 Department of Biostatistics, University of North Carolina Chapel Hill, Chapel Hill, North Carolina, United States of America; 9 Boston VA Research Institute, Boston, Massachusetts, United States of America; 10 Brigham Women’s Hospital Division of Aging, Harvard Medical School, Boston, Massachusetts, United States of America; Indiana University Richard M Fairbanks School of Public Health, UNITED STATES

## Abstract

In the current precision medicine era, more and more samples get genotyped and sequenced. Both researchers and commercial companies expend significant time and resources to reduce the error rate. However, it has been reported that there is a sample mix-up rate of between 0.1% and 1%, not to mention the possibly higher mix-up rate during the down-stream genetic reporting processes. Even on the low end of this estimate, this translates to a significant number of mislabeled samples, especially over the projected one billion people that will be sequenced within the next decade. Here, we first describe a method to identify a small set of Single nucleotide polymorphisms (SNPs) that can uniquely identify a personal genome, which utilizes allele frequencies of five major continental populations reported in the 1000 genomes project and the ExAC Consortium. To make this panel more informative, we added four SNPs that are commonly used to predict ABO blood type, and another two SNPs that are capable of predicting sex. We then implement a web interface (http://qrcme.tech), nicknamed QRC (*for* **QR** code based **C**oncordance check), which is capable of extracting the relevant ID SNPs from a raw genetic data, coding its genotype as a quick response (QR) code, and comparing QR codes to report the concordance of underlying genetic datasets. The resulting 80 fingerprinting SNPs represent a significant decrease in complexity and the number of markers used for genetic data labelling and tracking. Our method and web tool is easily accessible to both researchers and the general public who consider the accuracy of complex genetic data as a prerequisite towards precision medicine.

## Introduction

Genomic data is being accumulated at an incredible rate. It is projected that approximately one billion people will be whole genome sequenced within the next decade[1]. With a cost easily below $100, genotyping arrays that target single nucleotide polymorphisms (SNPs) will increase this rate exponentially. Many studies, such as the UK biobank project[2] in United Kingdom, the VA million Veteran program[3] in United States, the China Kadoorie Study[4] in China and United Kingdom, have taken advantage of these cost-effective arrays to genotype samples up to ~500,000. These large cohorts are not anomalies, with the Kaiser Perch Program on Genes, Environment, and Health[5] and the and TOPMed[6], building cohorts of similar size. Outside of the research field, direct-to-consumer genetic testing has exploded, with companies claiming to have genotyped more than a million individuals (for example, http://www.23andme.com).

However, with this plethora of genetic data comes errors. Hu et al. report an average rate of error for sample mix-up between 0.1% to 1%,[7] suggesting that between 500 to 5,000 samples are probably mislabeled for a large study such as the UK Biobank Study. A significant amount of research has been devoted to reducing these errors and improving the quality control. These strategies range from devoted and detailed outlines of quality control procedures[8] to matching sets of significant markers for sample tracking. All of these methods require a significant amount of expertise and time to implement, making them a drain on limited resources.

Individual identifications by SNP analysis require generation of a panel of SNPs that together give an extremely remote probability that two individuals would have the same DNA profile. Previously, a universal panel of 92 SNPs was developed for individual identification[9]. Another panel used 75 SNPs for Eastern Asian populations[10]. A recent simulation study showed that only 60 optimized SNPS are required to differentiate individuals in the global population[7]. In this study, we describe a solution that is accurate, unique, and easy to use. Our proposed solution uses 80 identified SNPs that are shared across widely used genome-wide genotyping arrays. To increase the accessibility and easiness of use, we develop on online platform to extract the genetic data and encode it as a quick response (QR) code. QR codes have the advantage of being a robust method for encoding information and can be read with any image capture devise such as a smart phone. Liu et al. previously compared 53 different types of one-dimensional and ten two-dimensional barcode symbologies and found that the QR code has the largest coding capacity and relatively high compression rate, allowing for easy expansion if necessary[11]. Our website, nicknamed QRC (*for* **QR** code based **C**oncordance check), provides an easy to use web based interface for extracting the 80 markers from uploaded genotype data, encoding the markers as a QR code, and comparing the concordance of multiple QR codes. This methodology can easily be expanded to be used by professionals in the genetic field.

## Methods

### Identification of ID SNPs

To generate our list of fingerprinting SNPs, we first obtained a list of bi-allelic autosomal SNPs that overlap in eight widely used genotyping arrays: three Affymetrix arrays including Axiom Biobank Array, Axiom UK biobank Array, and the newly announced Axiom Precision Medicine Research Array (PMRA) (http://www.affymetrix.com/catalog); three Illumina arrays including infinium-omniexpress-24-v1-2-a1 array, Illumina HumanExome-12v1-2 array, and the newly announced Global Screening array (GSA) (http://www.illumina.com/techniques/microarrays), as well as two direct-to-consumer (DTC) arrays (23&Me and Genes for Good). The resulting list is then selected again to ensure at least moderate frequencies across global populations. Specifically, we select SNPs with minor allele frequency (MAF) over 0.25 in each of the five global sub-populations presented in the 1000GP project, so that the selected are not only available in major genotyping arrays, but are also common in global populations. The five sub-populations are: European (EUR), African (AFR), Native American (AMR), Eastern Asian (EAS), and Southern Asian (SAS). The MAF is based on data from the 1000 genomes project (1000GP)[12] (freezing date 20130502) and the Exome Aggregation Consortium (ExAC)[13] (release 0.3.1). The former includes whole genome sequencing data from 2,504 individuals of diverse ancestry while the latter whole exome sequencing data from over 60,000 individuals.

The results are further pruned by removing A/T and C/G SNPs and SNPs annotated as pathogenic or likely pathogenic as reported by ClinGen database[14]. The final selection process limits to those SNPs that are not marginally dependent with each other, i.e., are in linkage disequilibrium (LD). To be very conservative, we pick only one SNP from any 10MB region on the genome. The SNP for a given region was selected as having the highest overall MAF over the remaining SNPs. Across the whole genome this resulted in 74 SNPs that satisfy our filtering criteria. This number slightly exceeds the theoretical number of 60 required to uniquely distinguish the global population[7]. To make this panel verifiable on its own when there is only one genetic dataset, we added four single nucleotide variants (SNVs) that are commonly used to predict ABO blood type: (1). exon-6 deletion rs8176719 for O1 type; (2). rs41302905 for O2 type; (3). rs8176746 for B type[15, 16]; (4). rs56392308 for A2 subtype[17]. We further added two SNPs that are capable of predicting sex: rs12743401, rs12734338. These two SNPs are aligned to both chromosomes 1 and Y, therefore, heterozygosity in male is actually a detection of two regions, one on chromosome 1 and the other on chromosome Y [18, 19]. The resulting total number of 80 SNPs were tested to confirm that they could uniquely label a large cohort. We used the UK Biobank (N ~150,000) as our test cohort. The genotypes of fingerprinting SNPs was extracted and tested for uniqueness using PLINK[20].

### Comparing the concordance of ID SNPs through QR codes

We then developed a web based application (http://qrcme.tech) that can extract the genotypes for these fingerprinting SNPs from raw genotype datasets such as those from 23&Me and then generate QC codes. To create a QR code, we first generate a string in the format of “1AA2AC3—”, where 1,2,3 are the index of 80 SNPs and the two digit letters are the genotype of SNPs at that position. Missing data is represented by “-”. Then, this string, without indices, is encoded into a QR code using the open source Zebra Crossing barcode image processing library (https://github.com/zxing/zxing/). This same library is used to decode a QR image back to the original text string. To compare QR Codes, we first decode both images, and compare the 80 SNPs values from the decoded strings. A match includes five scenarios: (1) a perfect match such as “AG” vs. “AG”, (2) a permuted match such as “AG” vs. “GA”, (3) an opposite strand match such as “AG” vs. “TC”; (4) an “AC” vs.”TG” match (all permutations); (5) an “AG” vs. “TC” match (all permutations). All other conditions are considered a mismatch, with missing data reported separately.

For those who are interested in deriving their own list of ID SNPs, we have also made it easy to accomplish through our QRC website. It takes a list of SNPs in CHR:POS format and compares it with a reference file that includes allele frequencies of 1,388,180 biallelic variants existing in both 1000GP and ExAC. Then it generates a list of independent SNPs with high allele frequencies across all major sub-populations, based on user specified MAF cutoff and region size threshold.

## Results

### Identification of ID SNPs

**Fig 1:**
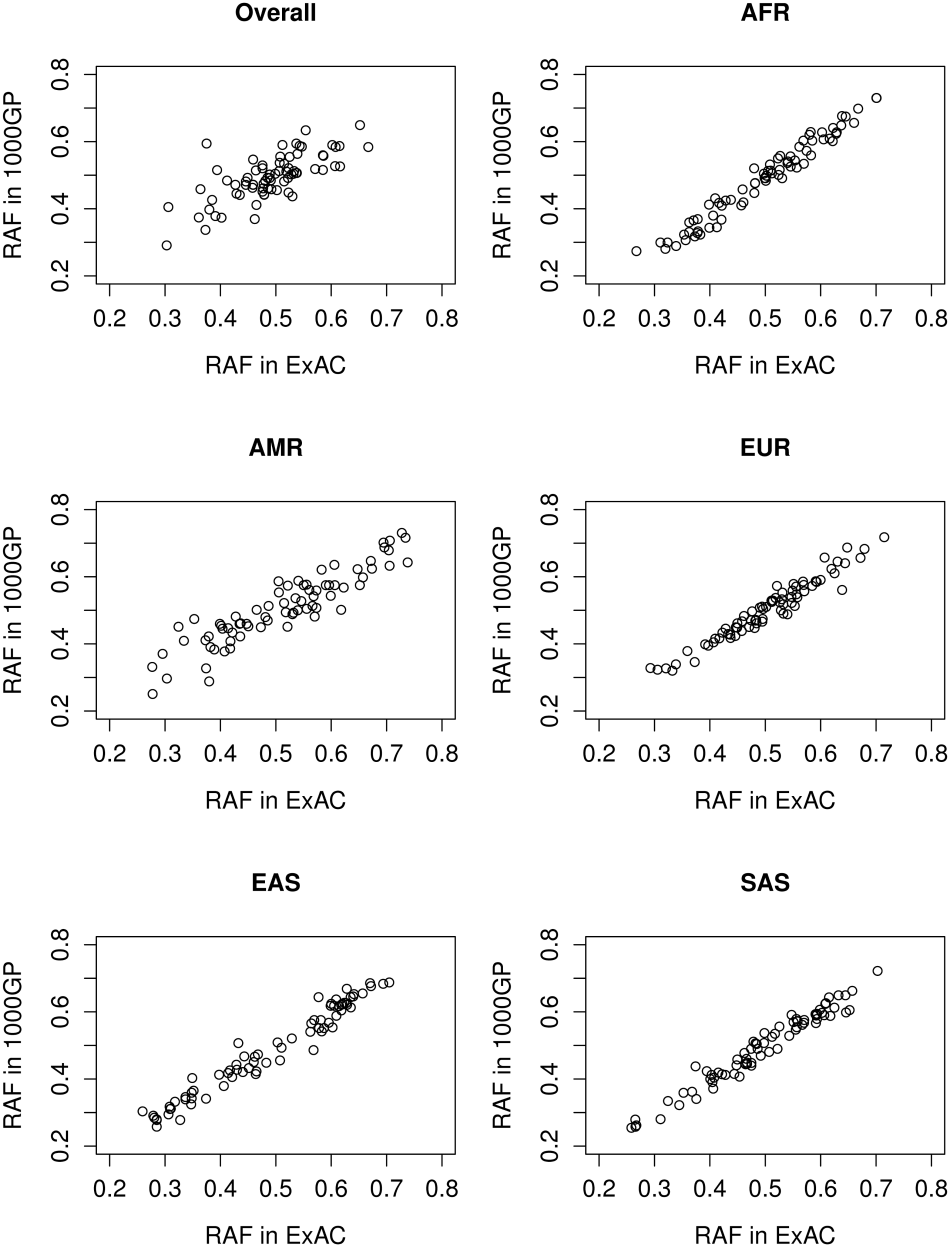
Reference allele frequency of the selected 80 SNPs. Reference allele frequency across the five major population groups (African: AFR, European: EUR, Native American: AMR, Eastern Asian: EAS and Southern Asian: SAS) and overall as reported by 1000GP and ExAC. Y-axis is the RAF in ExAC.

Through a series of selections, we have identified 74 SNPs across the whole genome that uniquely identify an individual across the global population. To make this list of SNPs more informative and unique, we further included four SNPs for predicting ABO blood type and two SNPs for predicting sex. Therefore, there is a total of 80 SNPs are included. Table 1 shows the overlapping of SNPs across eight major genotyping arrays. The upper diagonal numbers are the numbers of overlapping SNPs for each corresponding pair. The lower diagonal numbers (shown in italicized font with an underline) are the cumulative numbers of overlapping SNPs for each corresponding pair. For example, there are 865,720 SNPs in Axiom PMRA array, among which 272,701 are also present in Axiom UK Biobank array. Among the 272,701 SNPs, 172,088 are also in Axiom Biobank array. And among the 172,088 SNPs, 39,292 are also on Illumina GSA array. Eventually, 3,239 SNPs are shared across all eight arrays and 74 are independent. The details for these 74 fingerprinting SNPs are listed in Table 2. The reference allele and reference allele frequency (RAF) was based on the human reference genome^15^. These 74 SNPs span 20 autosomes, excluding chromosomes 15 and 21. They overall MAF is all greater than 0.3, based on the 2,504 multi-ethnical individuals in 1000GP. There is at least 10MB separating SNPs with the average distance being 37.4MB reducing the possibility of linkage between SNPs. Additionally, these SNPs have no reported pathogenic or likely pathogenic association according to the ClinGen database meaning these SNPs reveal no information regarding disease risk. Fig 1 shows the RAF between 1000GP and ExAC for these 74 SNPs.

**Table 1:** Cross tabulation of bi-allelic autosomal SNPs across eight arrays.

	**Axiom PMRA**	**Axiom UK Biobank**	**Axiom Biobank**	**Illumina GSA**	**Omni Express**	**23&Me**	**Genes for Good**	**Exome Array**
**Axiom PMRA**	865,720	272,701	207,468	128,503	82,373	70,227	61,240	21,941
**Axiom UK Biobank**	272,701	800,194	359,529	289,548	103,360	91,747	103,139	65,910
**Axiom Biobank**	172,088	172,088	629,487	105,807	77,132	65,734	232,406	185,863
**Illumina GSA**	39,292	39,292	39,292	733,348	185,489	113,481	192,333	54,913
**Omni Express**	15,905	15,905	15,905	15,905	693,518	303,948	253,917	18,683
**23&Me**	10,478	10,478	10,478	10,478	10,478	510,550	128,062	15,684
**Genes for Good**	8,385	8,385	8,385	8,385	8,385	8,385	540,551	233,277
**Exome Array**	3,239	3,239	3,239	3,239	3,239	3,239	3,239	238,468

The numbers highlighted in grey along the diagonal line are for each individual SNP panel. The upper diagonal numbers are the numbers of overlapping SNPs for each corresponding pair. The lower diagonal numbers (shown in italicized font with an underline) are the cumulative numbers of overlapping SNPs for each corresponding pair. For example, for the second column, there are 865,720 SNPs in Axiom PMRA array, among which 272,701 are also present in Axiom UK Biobank array, among the 272,701, 172,088 are also in Axiom Biobank array, and among the 172,088, 39,292 are also on Illumina GSA array, etc; and eventually, 3,239 are shared across all eight arrays.

**Table 2:** List of fingerprint SNPs.

#	Chr	Pos (b37)	rsID	Ref	Alt	RAF	#	Chr	Pos (b37)	rsID	Ref	Alt	RAF
**1**	1	7,202,190	rs970973	T	C	0.539	**38**	8	1,514,009	rs2301963	C	A	0.477
**2**	1	34,071,525	rs1874045	C	T	0.571	**39**	8	30,973,957	rs1800392	G	T	0.446
**3**	1	110,998,854	rs7514102	G	A	0.435	**40**	8	121,228,679	rs4870723	A	C	0.512
**4**	1	161,479,745	rs1801274	A	G	0.479	**41**	8	143,761,931	rs2294008	C	T	0.306
**5**	1	183,542,387	rs2274064	T	C	0.489	**42**	9	4,576,680	rs301430	T	C	0.364
**6**	1	203,194,186	rs2297950	C	T	0.303	**43**	9	15,784,631	rs1539172	A	G	0.478
**7**	1	225,534,219	rs7527925	T	C	0.476	**44**	9	116,136,198	rs1043836	C	T	0.615
**8**	1	248,039,713	rs3811445	A	G	0.608	**45**	9	133,927,878	rs10901333	A	G	0.459
**9**	2	26,804,247	rs935172	T	C	0.547	**46**	10	6,001,696	rs3136618	C	T	0.507
**10**	2	101,638,888	rs3739014	A	G	0.607	**47**	10	30,316,208	rs2185724	T	C	0.373
**11**	2	113,309,473	rs1545133	C	T	0.523	**48**	10	99,498,234	rs3818876	G	A	0.53
**12**	2	138,420,996	rs10206850	A	G	0.543	**49**	10	124,610,027	rs1891110	G	A	0.528
**13**	2	191,301,368	rs9646748	A	G	0.485	**50**	10	134,748,331	rs12781609	C	T	0.402
**14**	2	207,041,053	rs3732083	T	C	0.458	**51**	11	14,246,296	rs1025412	G	A	0.515
**15**	2	237,149,941	rs6756597	C	T	0.479	**52**	11	33,065,394	rs1064005	C	T	0.38
**16**	3	14,755,572	rs6765537	A	G	0.391	**53**	11	73,785,326	rs4453265	T	C	0.476
**17**	3	52,727,257	rs2289247	G	A	0.429	**54**	12	16,397,734	rs1852450	C	A	0.489
**18**	3	100,963,154	rs571391	G	A	0.652	**55**	12	58,162,739	rs703842	A	G	0.385
**19**	3	122,259,606	rs9851180	T	C	0.538	**56**	12	125,467,158	rs11558556	C	T	0.361
**20**	3	193,209,178	rs6788448	T	C	0.427	**57**	13	33,703,656	rs495680	T	C	0.585
**21**	4	42,639,186	rs898500	A	G	0.481	**58**	13	50,141,345	rs4942848	G	A	0.616
**22**	4	79,443,850	rs931606	G	A	0.519	**59**	14	23,299,135	rs1135641	G	T	0.464
**23**	4	187,120,211	rs13146272	C	A	0.585	**60**	14	73,138,189	rs1060570	C	A	0.449
**24**	5	1,065,399	rs737154	C	T	0.525	**61**	14	101,350,298	rs3825569	T	C	0.506
**25**	5	52,193,287	rs1531545	C	T	0.554	**62**	16	4,751,045	rs863980	C	T	0.533
**26**	5	73,339,114	rs285599	C	T	0.394	**63**	16	29,998,200	rs4077410	A	G	0.491
**27**	5	96,503,523	rs160632	C	T	0.586	**64**	16	56,995,236	rs1800775	C	A	0.459
**28**	5	150,943,085	rs2304054	G	A	0.465	**65**	17	14,005,439	rs2159132	G	A	0.522
**29**	5	169,685,163	rs315717	C	T	0.508	**66**	17	33,749,546	rs2586514	A	G	0.602
**30**	6	31,610,686	rs1052486	A	G	0.499	**67**	17	57,963,537	rs1292053	A	G	0.446
**31**	6	129,807,629	rs2229848	C	T	0.667	**68**	17	71,196,809	rs1026128	A	G	0.523
**32**	6	147,680,359	rs9390459	A	G	0.532	**69**	18	60,027,241	rs1805034	C	T	0.537
**33**	6	167,360,389	rs2236313	T	C	0.375	**70**	19	4,288,332	rs888930	A	G	0.412
**34**	7	33,282,577	rs7793096	G	A	0.502	**71**	19	17,394,124	rs2363956	T	G	0.486
**35**	7	99,757,612	rs3823646	G	A	0.537	**72**	19	49,658,367	rs3745298	C	T	0.459
**36**	7	141,672,604	rs10246939	T	C	0.476	**73**	20	52,786,219	rs2296241	G	A	0.492
**37**	7	156,762,248	rs12919	G	A	0.515	**74**	22	19,951,271	rs4680	G	A	0.462

The resulting 74 SNPs sorted by chromosome and position as reported by build 37 reference genome. The RAF is based on 1000GP.

### Comparing the concordance of ID SNPs through QR codes

**Fig 2:**
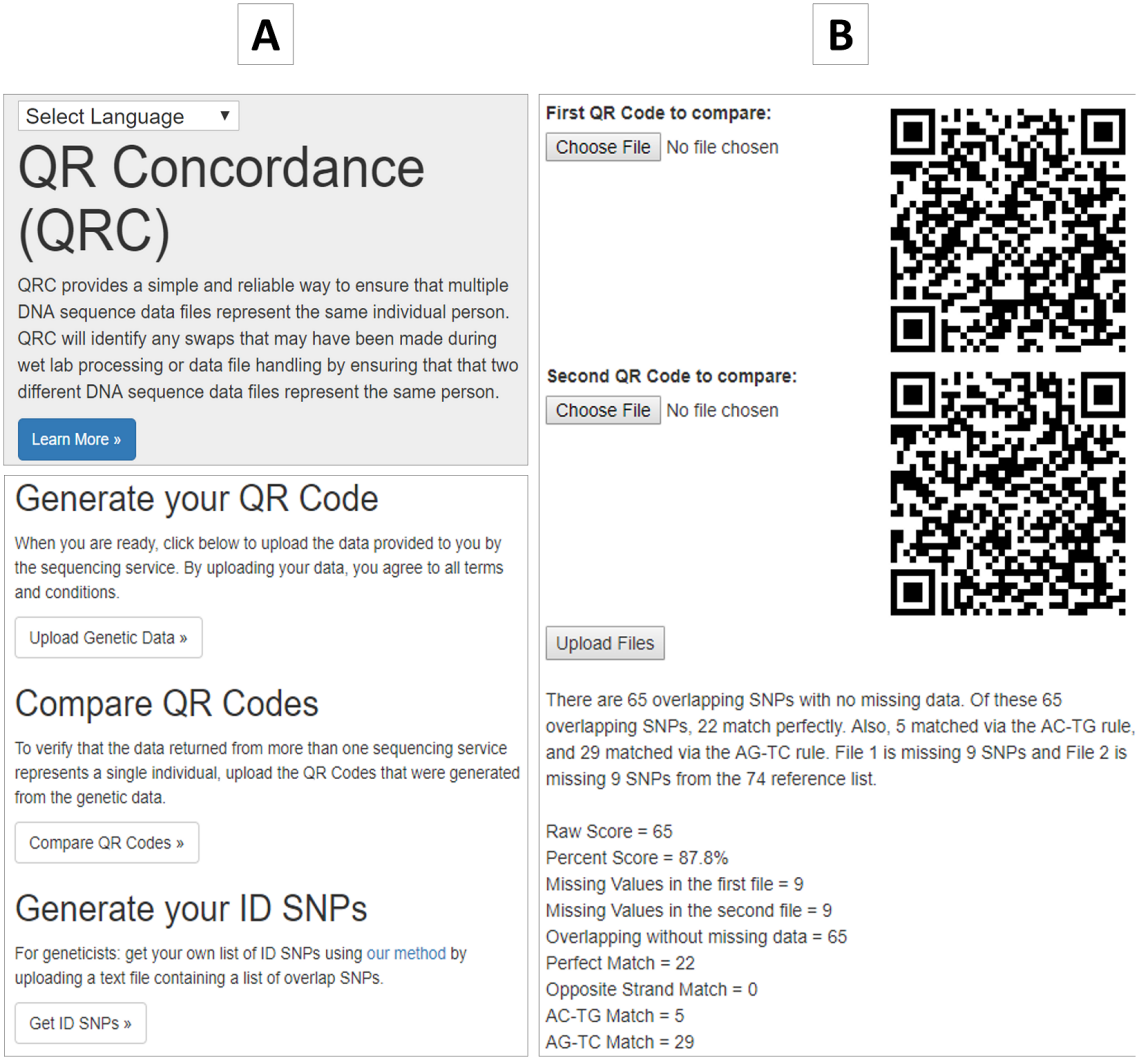
The QRC website interface. **A**. The interface allows a user to first upload genetic data to generate a QR code and save it into his local computer, and then compare any two QR codes for concordance check. Researchers could also generate their own ID SNPs. **B**. A sample report, based on genotype datasets for two different individuals. The report includes the number of missing SNPs and the overlap of non-missing SNPs and the type of matches.

As shown in Fig 2A, our web tool allows users to do three things: 1. Generate one or more QR codes from one or more raw genotype datasets and save the QR codes locally; 2. Compare two QR codes to get a report on the concordance of the underlying genotype datasets; 3. Generate one’s own ID SNPs. This is primarily for those savvy users including researchers who prefer to generate their own ID SNPs instead of using the 80 SNPs that we derived. Fig 2B shows a example report. It is based on genotype datasets for two different individuals, therefore, the concordance is low. The report includes the number of missing SNPs and the overlap of non-missing SNPs and the type of matches.

## Discussion

Short tandem repeat (STR) markers have been routinely used for genetic fingerprinting forensic settings, because of the large number of alleles within various populations[21]. However, STR does have disadvantages, including high mutation rate, lack of high-throughput technologies, and the need for large amplification products and therefore limits the use of degraded samples.[22] In this manuscript, we have presented a method for creating a list of identifying SNPs. This method uses a series of selections, the first being identifying overlapping SNPs across eight genotyping arrays. The results are further selected by requiring a minimum MAF value above 0.25 across the five major continental groups. Additional selections result in just 80 SNPs that uniquely identify individuals across the global population. We have confirmed this uniqueness in the large publicly available genetic database, the UK biobank. This same procedure can be implemented in other settings to create similar lists that fit a given need.

Our identified list of 80 SNPs, has the practical application of reducing the number of SNPs used for comparison in the tracking of genetic data through the genotyping pipeline. Genotyping vendors currently use their own list of SNPs for tracking, with Affymetrix reportedly using over 300 markers for sample tracking. Our lower number of markers results in faster comparisons leading to savings in time and possibly cost, especially over millions of samples as reported by 23&Me. We further implemented the QRC web server (http://qrcme.tech). The simple and easy to use graphical interface allows a user to upload a genetic data set, which is parsed for the genotypes at the 80 SNPs. The results are then encoded as a QR code that can be attached to a data set. QR codes from different data sets can also be compared, leading to a check across commercial genotyping companies. This feature has already been implemented in addition to coding and decoding QR codes. This methodology can be easily expanded to be used by professionals in the genetic field.

It is our goal to come up with a most parsimonious list of SNPs to uniquely identify any single person across the globe, through genetic data. However, our purpose is to encode this subset of genetic data into a QR code so that a non-geneticist could use an easy interface to check the concordance of one data with another, not for purposes such as forensic testing or paternity testing. Therefore, some level of uncertainty is tolerated. We further added SNPs that could be used to predict ABO blood type and sex, therefore one genotypic data alone could still provide some useful information for one to validate the data to some extent. It is our hope that the genetic community will work together to identify a robust method and agree upon an omnibus list of SNPs that could be used through user friendly interface like what is presented in QRC.
